# Live bacterial supplementation for improving treatment response in metastatic renal cell carcinoma

**DOI:** 10.1002/ctm2.948

**Published:** 2022-07-28

**Authors:** Luis Meza, Ameish Govindarajan, Matthew Feng, Sumanta K. Pal

**Affiliations:** ^1^ City of Hope Comprehensive Cancer Center Duarte California USA; ^2^ Pomona College Claremont California USA

1

It is estimated that about 79 000 new cases of kidney cancer will be diagnosed in 2022 in the United States, with varying rates of progression to metastatic disease.^1^ Although many new treatment options have become available in the last decade, 5‐year relative survival for patients with metastatic disease remains at a grim 14%.^2^ Among these new treatments are checkpoint inhibitors (CPIs), which work by targeting the cytotoxic T‐lymphocyte‐associated antigen 4 (CTLA‐4) and the programmed death‐1/programmed death‐ligand 1 (PD‐[L]1) axes. However, despite the potential promise for cure that these agents offer, around 20% of patients will present with progressive disease as best response.^3^ This has led to increased interest in identifying interventions that could meaningfully potentiate the effect of immunotherapy in patients with metastatic renal cell carcinoma (mRCC).

In recent years, researchers have been devoted to studying the role of the gut microbiome in cancer with many studies suggesting that it influences the response to CPIs. Through the evaluation of stool microbiome, multiple bacterial species have been implicated in the development of a more robust response to CPIs in mRCC. Notably, increased relative abundances of *Akkermansia* spp. and *Bifidobacterium* spp. have been associated with this effect.^4^, ^5^ These results are bolstered by preclinical models showing that delayed tumour progression could be achieved by oral administration of *Bifidobacterium* spp.^6^

Additionally, a retrospective study by Tomita et al. showed that live bacterial supplementation could lead to a positive impact to treatment with ICP in patients with lung cancer.^7^ However, despite these cumulative results pointing towards the potential of microbiome‐based interventions, the lack of prospective, randomized data has precluded its translation into a change in clinical practice.

In this context, our group carried out a phase I clinical trial evaluating the effect of live bacterial supplementation with CBM588 in patients receiving standard of care treatment with CPIs for mRCC.^8^ CBM588 is a live bacterial supplement comprised of *C* *lostridium butyricum*. This is a butyrate‐producing, gram positive, spore forming bacteria that has been shown to increase the abundance of *Bifidobacterium* spp. in the gut. In this study, 30 patients were randomised in a 2:1 ratio to receive nivolumab (a PD‐1 inhibitor) and ipilimumab (a CTLA‐4 inhibitor) in combination with CBM588, or alone (Figure 1).

**FIGURE 1:**
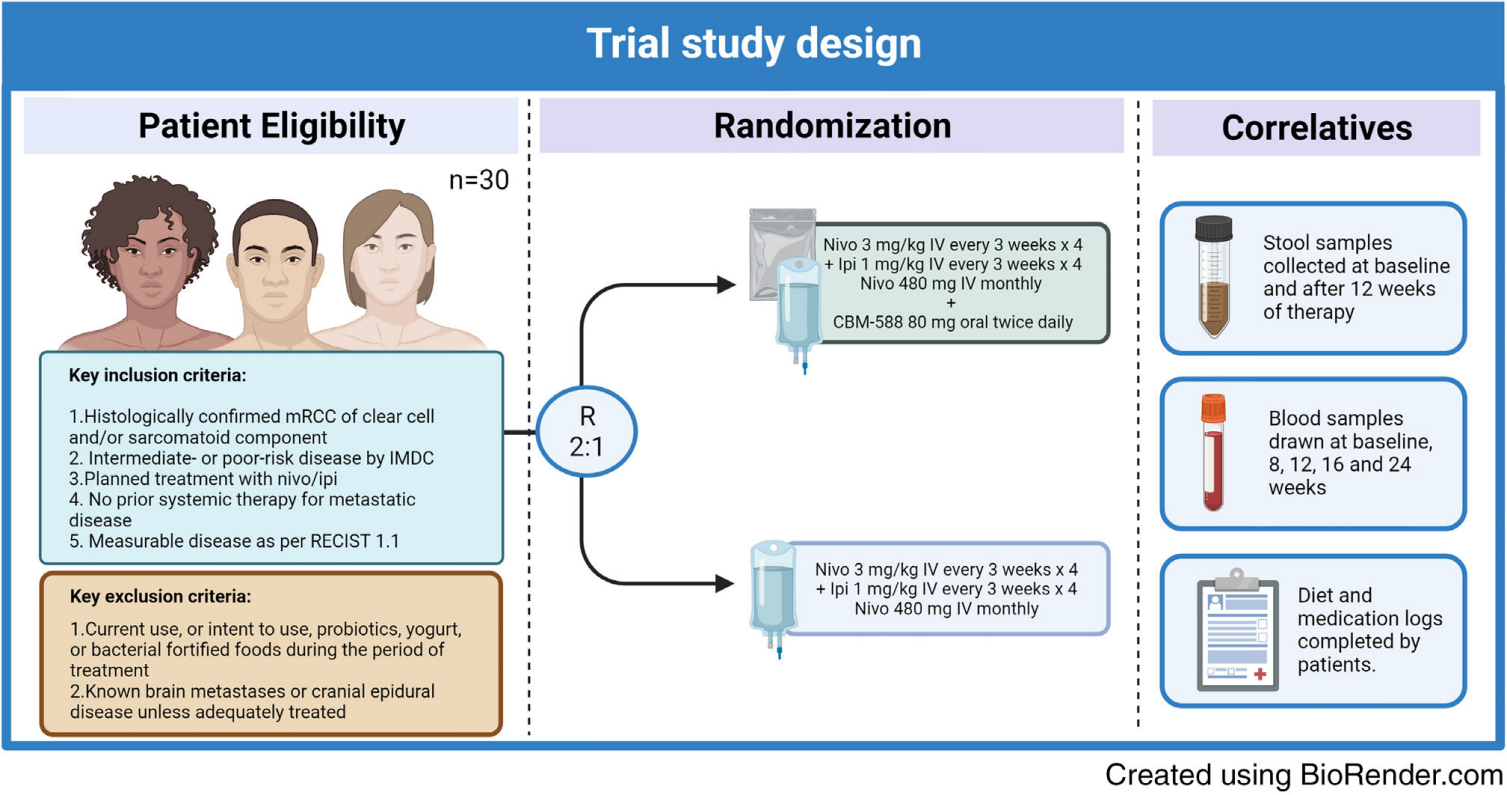
Summary of the study design

Despite the robust biological rationale behind the study, the study did not meet its primary endpoint of characterising the effect of CBM588 on the relative abundance of gut microbial populations and specifically *Bifidobacterium* spp. A potential explanation for this could be the need of a higher dose of CBM588 to evoke a significantly higher abundance of *Bifidobacterium* spp across the intervention cohort. However, it is interesting to note that patients receiving CBM588 who responded to treatment did have an increased relative abundance of *Bifidobacterium* spp. Furthermore, the use of whole metagenome sequencing instead of 16S ribosomal RNA gene amplicon sequencing enabled us to identify changes in metabolic pathways associated with CBM588 supplementation which are detailed in the main publication.

Perhaps the most notable finding of our study was the clear progression‐free survival (PFS) advantage in the patients receiving CBM588 over those receiving standard of care alone (12.7 vs. 2.5 months, hazard ratio 0.15, 95% CI 0.05–0.47, *P*  <  .001). Response rate was also markedly higher with the addition of CBM588 (58% vs. 20%, *P *= .06). In addition to this, safety of both regimens appeared to be comparable, with 50% and 52% of patients developing grade 3 or 4 adverse events, for the control and intervention arm, respectively. The clinician could question the seemingly underperforming control arm, especially when compared to the pivotal trial that led to the approval of the nivolumab–ipilimumab combination (PFS 11.6 months).^3^ This could be explained by the small‐sample size, unmeasured confounders, or perhaps more significantly by the fact that patients in the control arm were probiotic restricted.

Based on our results and the existing array of retrospective and preclinical evidence available, bacterial supplementation with CBM588 could represent a way of augmenting efficacy of CPIs in patients with mRCC without incurring additionally toxicity. However, the results of this experience should be interpreted with great caution given the limited sample size.

Although many studies to date have identified overlapping bacterial species in the gut to be influential in systemic treatment, such as *Bifidobacterium* spp., one of the biggest challenges remains constructing standard operating technical procedures for the analysis of the gut microbiome. This is exemplified by the mostly heterogeneous species of bacteria reported across studies, the associations of which remain modest. More preclinical and clinical studies are needed to develop a deeper collective understanding of the mechanisms behind the influence of the gut milieu in anti‐tumoral immunity. Admittedly, to fully understand the changes in the setting of disease, it would be necessary to identify what constitutes a “normal” microbiome and what patient intrinsic characteristics could affect it (e.g., geographic location, diet, etc.). This would ideally be achieved through population‐wide studies, which, although labour‐ and cost‐intensive, would bear significant fruit. Studies evaluating the microbial composition of sites beyond the gut are currently ongoing. The assessment of the intra‐tumoral microbiome, for example, could also provide insights into the complex interplay between microbial species and intra‐tumoral immunity.^9^

Finally, although our experience represents a forward stride in the development of strategies to safely augment the activity of CPIs, larger prospective validation is needed for this strategy to earn a role in routine clinical practice. Moreover, a remaining question is whether these clinical observations will be maintained in the context of the rapidly evolving treatment landscape for mRCC. To help answer this, a new clinical trial is underway that will evaluate the effect of CBM588 supplementation in patients receiving nivolumab in combination with cabozantinib, a receptor tyrosine kinase inhibitor (TKI) with a broad range of targets including MET, AXL and VEGF^10^ (NCT05122546).

## CONFLICT OF INTEREST

The authors declare that there is no conflict of interest that could be perceived as prejudicing the impartiality of the research reported.
